# Antibiotic-Eluting Envelopes for the Prevention of Cardiac Implantable Electronic Device Infections: Rationale, Efficacy, and Cost-Effectiveness

**DOI:** 10.3389/fcvm.2022.855233

**Published:** 2022-03-28

**Authors:** Vassil Traykov, Carina Blomström-Lundqvist

**Affiliations:** ^1^Department of Invasive Electrophysiology, Acibadem City Clinic Tokuda University Hospital, Sofia, Bulgaria; ^2^Department of Medical Science, Uppsala University, Uppsala, Sweden; ^3^Department of Cardiology, Faculty of Medicine and Health, School of Medical Sciences, Örebro University, Örebro, Sweden

**Keywords:** cardiac implantable electronic device, infection, pacemaker, cardiac resynchronization therapy, implantable cardioverter defibrillator, antibiotic eluting envelope, cost-effectiveness

## Abstract

Infections related to cardiac implantable electronic devices (CIED) are associated with significant morbidity and mortality. Despite optimal use of antimicrobials and other preventive strategies, the incidence of CIED infections is increasing over time leading to considerable costs to the healthcare systems. Recently, antibiotic-eluting envelopes (AEEs) have been introduced as a promising technology to prevent CIED infections. This review will address the current evidence on stratification of CIED infection risk, present the rationale behind AEE, and summarize the currently available evidence for CIED infection prevention as well as demonstrate the cost-effectiveness of this novel technology.

## Introduction

Since the initial experience with electronic pacemakers in the late 1950s and the introduction of implantable cardioverter-defibrillators in the 1980s, cardiac implantable electronic devices (CIEDs) have become routine therapy of numerous arrhythmias and conduction disturbances. The numbers and complexity of CIED implantations continue to rise worldwide (1), especially with the introduction of cardiac resynchronization pacemakers (CRT-P) and defibrillators (CRT-D) (2) which has been accompanied by an increasing rate of complications. Device infection is an important factor for increased morbidity and mortality among CIED recipients (3). The rate of CIED infections has been shown to increase over the years (1, 4). Among the possible causes are increasing complexity of implanted devices, increasing comorbidities, and longer life expectancy with the need for multiple generator replacements and lead revisions.

Although various preventive strategies have been proposed to reduce these serious and costly CIED complications (5) there is a significant discrepancy in the implementation of the different preventive strategies worldwide (6). The rules of antisepsis and preoperative antibiotic prophylaxis have been shown to be highly effective and are recommended by consensus papers and guidelines (5, 7). The introduction of subcutaneous ICDs and leadless pacemakers may also contribute to a reduction of CIED infections but are applicable only in a selected patient population. The implantation of antibiotic-eluting envelopes (AEE) currently presents a promising strategy to prevent CIED infections in patients at risk for device infections including those not suitable for the currently available leadless or subcutaneous technology. As such, AEE use has been recommended by recent guidelines and consensus statements (5, 7).

The aim of this review is to summarize the currently available data on risk stratification of CIED infections, present the rationale behind AEE, and summarize the available evidence on the benefit of AEEs for the prevention of CIED infection including its cost-effectiveness.

## Epidemiology and Microbiology of CIED Infections

Device-related infections, ranging from 1 to 7% depending on the type and complexity of the implantation (2, 8, 9), are among the most devastating complications of CIED implantations resulting in significant morbidity and mortality (3, 10). Data from the US National Inpatient Sample Database encompassing 4,144,683 device-related procedures from 2000 until 2012 demonstrated a significant rise in the infection rates over time from 1.45% to 3.41% with the highest increase for CRT-P/D devices (1). This contrasts with recent randomized studies reporting much lower infection rates in the range of 0.6–1.3% (3, 9, 11). In addition, very recent real-life, nonrandomized data demonstrates infection rates comparable to that reported in the randomized trials (12, 13). A study by Lee et al. reported 7.2% in-hospital mortality and 25.3% mortality at 1 year (3) among 387 patients following lead extraction for CIED infection. In contrast, more recent retrospective data from a single center study demonstrate lower 30-day mortality rates following transvenous lead extraction (due to CIED infection in 93% of the studied population) (14). The trend for increased mortality despite successful infection eradication was preserved at 3 years as reported by Sohail et al. (10).

There are two basic mechanisms of CIED infections: contamination during implantation (15) and bloodstream infection (16). The most common manifestation of CIED infection is pocket infection (9, 16). In the most typical clinical scenario (due to contamination) the pocket infection develops in the first 12 months following implantation although skin erosion late after implantation can also be seen (16, 17). The infection spreads along the leads and eventually causes systemic infection resulting in device-related endocarditis. Bacteremia due to remote infectious foci (e.g., as a result of contaminated vascular catheters, surgical site infection, septic thrombophlebitis, etc.) leads to direct lead seeding which later progresses to systemic infection usually leaving the pocket intact.

The microbiology of CIED infections includes mainly Gram-positive bacteria (70–90% of the isolates) some of which are normally non-pathogenic. The latter are most commonly coagulase-negative staphylococci (mainly *Staphylococcus epidermidis*). *Staphylococcus aureus* is another commonly isolated bacterium in cases of pocket infection (especially in early cases); it is also the most common cause of bacteremia (18–22). Methicillin-resistant staphylococci have been reported to be the underlying cause in almost half of all staphylococcal CIED infections (18). Gram-negative bacilli account for about 9% of the infections while fungi are rare (22).

## Identifying High-Risk Patients

The highest benefit from any preventive measure is projected to the population at highest risk. Therefore, estimating infection risk in each patient is of utmost importance to identify the CIED recipients where more aggressive preventive measures should be taken to reduce infection rate. Risk factors associated with higher CIED infection risk can be grouped into patient-related, procedure-related, and device-related (Table 1). Among the numerous patient-related factors, end-stage renal disease, prior CIED infection, advanced age, and preprocedural fever are associated with the highest infection risk (5, 23, 24). Procedural factors associated with greatest risk are early (<30 days) reintervention, procedure duration >1 h, pocket hematoma, and system revision/lead revision, upgrade or generator replacement (5, 23). Importantly, there is randomized data on the impact of hematoma formation on the CIED infection rate. The BRUISE CONTROL INFECTION study included 659 patients with CIED infection from the original study population and demonstrated that development of hematoma was associated with a more than 7-fold increased risk of infection (HR 7.7, 95% CI 2.9–20.5) within 1 year follow-up (27). Another very recent study analyzed the WRAP-IT population (*N* = 6,800 participants) and demonstrated a 2.2% incidence of hematoma 30 days after the implantation (26). The risk for CIED infection in patients with hematoma was 11-fold higher (HR 11.3, 95% CI 5.5–23.2) vs. uncomplicated cases. Device-related factors mainly include system size and complexity. Of these, implantation of CRT devices, the presence of more than two leads, and high energy devices are associated with increased infection risk, which has been corroborated by many studies. In one large Danish registry including 97,750 patients, 1,827 developed CIED infection. There was a significantly increased infection risk in patients with complex devices with hazard ratios (HR) of 1.26, 1.67, and 2.22 for ICD, CRT-P, and CRT-D systems (multivariate analysis, *P* < 0.002 for all entries), respectively, compared to conventional pacemakers (28). Higher infection rates were also reported in an observational study of patients implanted with ICD and CRT-D vs. pacemakers (29). Moreover, randomized data from the PADIT study demonstrated the importance of the procedure type as a risk factor for CIED infection (25). In that analysis, implantation of CRT and ICD as well as revisions/upgrades were associated with an increased risk for CIED infection OR 1.77 (1.09–2.87), 2.73 (1.72–4.31), and 4.01 (2.62–6.13), respectively (*P* < 0.02), for all comparisons. A very recent analysis of the randomized WRAP-IT trial provides firm evidence on the risk for CIED infection after a secondary procedure (30). Among risk factors, device type (CRT-P/D vs. ICD), number of previous procedures, history of atrial arrhythmia, geography (outside North America and Europe), procedure duration, periprocedural antithrombotic therapy, and device implant location were important risk factors.

**Table 1:** Major risk factors for CIED infections.

**Risk factors**	**Odds ratio**
**Patient-related factors**	
End stage renal disease	8.73
Prior CIED infection	7.84
Age ≥ 75 years	5.93
Fever prior to implantation	4.27
Immunosuppression	3.44
Renal failure	1.45*-3.02
COPD	2.95
NYHA class ≥ 2	2.47
Skin disorder	2.46
Immune compromise	2.28*
Malignancy	2.23
Diabetes mellitus	2.08
Heparin bridging	1.87
Congestive heart failure	1.65
Oral anticoagulation	1.59
**Device related factors**	
Epicardial leads	8.09
Abdominal pocket	4.01
CRT	2.73*
Two or more leads	2.02
ICD	1.77*
Dual chamber device	1.45
**Procedure-related factors**	
Reintervention <30 days	16.29
Procedure duration > 1 h	13.96
Haematoma	11.3^§^-4.95
Revision or upgrade	6.46-4.01
Lead repositioning	6.37
Replacement	4.93
Two or more prior procedures	3.43*
Inexperienced operator	2.85
Temporary pacing	2.31
Prior procedure	1.51*

*CIED, cardiac implantable electronic device; COPD, chronic obstructive pulmonary disease, CRT, cardiac resynchronization therapy; h, hour; ICD, implantable cardioverter defibrillator*.

*References marked with asterisks are randomized controlled trials*.

Figures taken from previously published non-randomized data by Polyzos et al. (23), Sławek-Szmyt et al. (24) AND to randomized data from Birnie et al. [*] (25) and Tarakji et al. [§] (26).

Development of risk score systems to stratify CIED recipients may be a promising tool for better identification of patients at low and high risk. One of the first attempts to create and implement a risk scoring system was by Mittal et al. who identified 7 clinical variables included in a risk score system ranging from 0 to 25 by using retrospective observational data from 2,981 patients (29). The infection risk increased significantly from the low-risk group (score 0–7, 1% infection rate) to the medium-risk group (score 8–14, 3.4% infection rate) and to the high-risk group (score ≥15, 11.1% infection rate). Another scoring system, including 10 clinical variables has been proposed by Sharriff et al. (31). It was later modified and was recently demonstrated to identify high CIED infection risk in 1,391 patients undergoing first-time implantation. (32). In this retrospective study Shariff score ≥ 4 was associated with more than three-fold increased risk of CIED infection–RR 3.20 (1.29–12.59), *P* = 0.029. Kolek et al. also proposed a scoring system consisting of several clinical variables known to be associated with CIED infection risk (33, 34). The recently developed PADIT risk score system (25) identified five independent predictors: prior procedure (P), age (A), depressed renal function (D), immunocompromised (I), and procedure type (T). The score, ranging from 0 to 15 points, was used to group patients into low (0–4 points), intermediate (5–6 points), and high (≥7 points) risk groups with hospitalization rates due to CIED infection of 0.51, 1.42, and 3.41%, respectively. The predictive value of the PADIT risk score has recently been validated in a large real-world dataset comprising 54,042 procedures where each unit increase in PADIT risk score was associated with 28% increase in infection risk (35). Very recently Boriani et al. have also introduced a scoring system (RI-AIAC score) based on real-life registry data including 2,675 patients (13). They have identified three major clinical characteristics associated with increased CIED infection risk and have created a 5-point scoring system. The latter was tested for predictive ability in the study population and was compared against the PADIT, Shariff and Kolek scores in that regard. Results demonstrated a modest predictive ability of RI-AIAC score with a C-index of 0.64 (0.52–0.75) and of PADIT score with a C-index of 0.64 (0.53–0.76) while the other two risk scores were not able to predict infectious outcome in this population.

## Antibiotic Eluting Envelopes: Technology

Early versions of AEEs consisted of non-absorbable polypropylene mesh, but this design was associated with significant pocket fibrosis and was therefore abandoned. There are currently two absorbable CIED envelope devices on the market. One of them (CanGaroo-G™, Aziyo Biologics, Silver Spring Inc, MD, US) is made from a decellularized and non-crosslinked extracellular matrix produced from porcine intestinal submucosa. That device does not possess antibiotic-eluting properties *per se* but can be impregnated with gentamycin prior to implantation (36). This ensures a peaking early antibiotic release and a stable level of the antibacterial agent for up to a week (36). Animal data has shown the lack of bacterial growth in device pockets inoculated with six different microbial species and exposed to gentamycin-impregnated AEEs. In this experiment, local gentamycin concentrations remained stable up to 7 days (37). The other commercially available envelope (TYRX™; Medtronic, Inc. Monmouth Junction, NJ, US) is made of a synthetic mesh of glycolide, caprolactone, and trimethylene carbonate absorbed in the body over a nine-week period. Both envelopes can stabilize the CIED in the pocket and reduce migration and erosion. However, only TYRX™ provides true antibiotic elution and will be discussed further on. The synthetic mesh is coated with an absorbable polyacrylate polymer that carries minocycline and rifampin and delivers them locally in the tissues over seven days. Both antimicrobials are active against Staphylococcus spp. (38). Rifampin has been shown to be active against *Staphylococcus epidermidis* in the biofilm where many other antibiotics are ineffective (39). The combination of minocycline and rifampin has been shown to have additive antibacterial effects on resistant bacteria such as methicillin-resistant *Staphylococcus aureus* (MRSA) (40). *In vitro* studies have shown the antimicrobial activity of TYRX™ against many bacteria such as MRSA and methicillin-sensitive *Staphylococcus aureus* and *Staphylococcus epidermidis* as well as *Escherichia coli* *(**41**)*. In an animal model of CIED implantation, TYRX™ effectively reduced infection after bacterial inoculation of the pocket (42). This AEE comes in two sizes: medium (designed for pacemaker implantations) containing 8.0 mg rifampin and 5.1 mg minocycline and large (designed for ICD implantation) with 11.9 mg rifampin and 7.6 mg minocycline (41).

## Evidence for the Benefit of Antibiotic Envelopes

The initial studies assessing efficacy of AEE were conducted with the older and nonabsorbable polymer design. One of the first publications including 624 patients undergoing PM, ICD, or CRT-D implantation showed low overall incidence of CIED infections: 0.48% [95% CI 0.17–1.40 (43). The lack of an active comparator makes it difficult to draw firm conclusions on AEE efficacy. A subsequent observational study demonstrated lower infection rates with AEE−0.4 vs. 3% in the control group (OR 0.13, 95% CI 0.02–0.95, *P* = 0.04) (33). This difference persisted in the propensity-matched cohort (OR 0.09, 95% CI 0.01–0.73, *P* = 0.02). The same group conducted another single center retrospective cohort study with similar outcome (34). After a minimum follow-up of 300 days, CIED infection rates were 0% for the TYRX™ group, 0.3% for the nonabsorbable AEE group, and 3.1% in the control group (*P* = 0.03 and 0.002 vs. controls, respectively). There was no difference in the infection rates between the two AEE groups. A larger retrospective observational study included 2,890 patients undergoing CIED implantation of whom 275 received an AEE (29). Propensity-matched analysis demonstrated a significantly lower infection rate at 6 months in the patients implanted with AEE 1.1 vs. 3.6% in the standard-of-care group (*P* = 0.048). The reduction in CIED infections was more expressed in the higher-risk population. In a single center observational study Shariff et al. also demonstrated significantly lower infection rates in AEE recipients−0 vs. 1.7% in the patients at similar risk not receiving the AEE (*P* = 0.006) (31). In contrast, one small retrospective study reported higher rates of major infections in AEE recipients: 5.4 vs. 1.1% in the standard-of-care group (*P* = 0.048) (44). However, the patients receiving AEE in this study had higher rates of chronic corticosteroid use, higher rates of replacement or revision, and were more frequently implanted with systems requiring >2 intra-cardiac leads. The Citadel and Centurion studies represent two multicenter prospective non-randomized registries enrolling patients undergoing CIED replacement or upgrade of an ICD (Citadel) or CRT (Centurion) with the use of a non-absorbable AEE (45). Among the studied population major CIED infection occurred in five patients (0.4%), significantly lower than the benchmark infection rate of 2.2% for these high-risk groups (*P* = 0.0023). A very recent two-center observational cohort study included 1,943 patients with CRT undergoing reoperation for replacement, upgrade, or revision who were followed up for a maximum of 2 years (46). An AEE was implanted in 736 patients (38%) with significantly more risk factors for CIED infection. The risk for CIED infection necessitating system extraction was reduced by 48% in the patients receiving an AEE (HR 0.52, 95% CI 0.30–0.90, *P* = 0.021).

The only randomized trial assessing the benefit of AEE in patients undergoing device implantations is the WRAP-IT trial, which included 6,983 patients randomized to AEE vs. standard of care (41). The primary endpoint was a major CIED infection in the 12 months following the operation. Patients included were those with increased risk of CIED infection: 1. Implantation of a *de novo* CRT-D; 2. Generator replacement or an upgrade of a previous implanted PM, CRT-P, ICD, or CRT-D; and 3. Pocket revision of an existing PM, CRT-P, ICD, or CRT-D. Certain patients with very high risk were excluded (e.g., those with previous pocket intervention in the previous 365 days, patients on dialysis on chronic immunosuppressive therapy, or those with previous CIED infection within 12 months). The study demonstrated a 40% reduction in major infections occurring in 0.7% of patients receiving TYRX™ vs. 1.2% in controls (HR 0.60, 95% CI 0.36–0.98, *P* = 0.04) (9). The positive outcome was entirely driven by the lower rate of pocket infections which comprised 75% of all major events–0.4 vs. 1% in the control group (HR 0.39, 95% CI 0.21–0.72). Subgroup analysis demonstrated a significant reduction in major CIED infection in patients receiving high-power devices (ICD and CRT-D) (HR 0.51, 95% CI 0.29–0.90); no difference was observed in the group receiving low-power devices (CRT-P and PM) (HR 1.02, 95% CI 0.236–2.02). The benefit of TYRX™ was sustained during longer term follow-up (mean 21 ± 8.3 months) with a persistent reduction in CIED infections to 1.3% in the AEE group vs. 1.9% in the control group (HR 0.64, 95% CI 0.41–0.99) (47). Further analyses of the WRAP-IT population demonstrated a more than 11-fold higher risk of major CIED infection in patients with pocket hematoma and without the AEE (26). In patients who received the AEE and later developed pocket hematoma the risk was 82% lower (HR 0.18; 95% CI 0.04–0.85%, *P* = 0.03) and the infection rate was comparable to those without hematoma.

In a recent meta-analysis summarizing six major observational and randomized studies comprising 11,897 patients (5,844 receiving the envelope) the AEE was associated with a 66% relative risk reduction of major CIED infections in high-risk patients (RR 0.34; 95% CI 0.14–0.86, *P* = 0.02) (48). A subgroup analysis including only high-risk patients demonstrated that the AEE use was associated with a 74% reduction in the relative risk for major CIED infection (RR 0.26, 95% CI 0.08–0.85, *P* = 0.03) and that there was no difference in the risk when the studies enrolling any risk patients were analyzed (RR 0.53, 95% CI 0.06–4,52, *P* = 0.56). A summary of all the available evidence on efficacy of AEE is presented on Table 2.

**Table 2:** Summary of the studies on efficacy of antibiotic-eluting envelopes.

**Authors**	**Year**	**Study design**	**Number of patients, AEE group/ comparator group**	**Envelope type**	**Follow-up duration**	**Patient population**	**Main results**	**Devices**
Bloom et al. (43)	2011	Retrospective	624/no comparator	Non-absorbable	1.9 ± 2.4 months	Consecutive initial implantation or revision/replacement procedures	Low overall incidence of CIED infections: 0.48%	PM, CRT-D, ICD
Kolek et al. (33)	2013	Observational	260/639	Non-absorbable	Minimum 90 days	Prospectively determined criteria for AE implantation	Significant benefit of AEE (OR 0.13, 95% CI 0.02-0.95, *P =* 0.04)	PM, CRT-D, ICD
Mittal et al. (29)	2014	Retrospective	275/275 (propensity matched controls)	Non-absorbable	Minimum 6 months	Single centre study on initial implantations, generator replacement or system upgrade	Lower infection rates in the AEE group vs controls: 1.1% vs. 3.6% (*P < * 0.048).	PM, CRT-D, ICD
Kolek et al. (34)	2015	Retrospective	488/636	Non-absorbable and absorbable	Minimum 300 days	> 2 risk factors for CIED infection: DM, CKD, OAC, chronic steroid use, prior CIED infection, > 3 trsv leads, early pocket reentry	Lower infection rates in both AEE groups vs controls: 0% and 0.3% vs. 3.1% (*P < * 0.03)	PM, CRT-D, ICD
Shariff et al. (31)	2015	Retrospective	365/1,111	Non-absorbable	Minimum 6 months	Initial CIED implantation, generator replacement or system upgrade	Lower infection rate in AEE groups vs standard-care group: 0% vs. 1.7% (*P =* 0.006)	PM, CRT-D, ICD
Hassoun et al. (44)	2017	Retrospective	92/92	Non-absorbable	Mean follow-up 9 months	CIED implantation at a single centre	• Higher rate of major CIED infection in AEE group vs standard-of-care group: 5.4% vs. 1.1% (*P =* 0.048). • Higher rates of revision/replacement (51.1% vs. 8.7%, *P =* 0.001); implantation of systems with >2 leads (42.4% vs. 29.3%, *P =* 0.03) and of chronic corticosteroid use in AE group vs controls.	PM, CRT-D, ICD
Henrikson et al. (45)	2017	Prospective	1,129/no active comparator	Non-absorbable	Minimum 12 months	Device upgrades, lead revisions or pulse generator replacements and high risk CIED infection patients	Major CIED infections less frequent in a high-risk AE group (0.4%) vs expected benchmark infection rate (2.2%) (*P =* 0.0023).	ICD/CRT-P/D
Tarakji et al. (9)	2019	RCT	3,495/3,488	Absorbable	12 months	High-risk patients undergoing CIED replacement, system upgrade, pocket or lead revision or initial implantation (some device types)	Major CIED infection Incidence 0.7% in AEE recipients vs. 1.2% in controls; 40% RRR (HR 0.60, 95% CI 0.36-0.98, *P =* 0.04). Effect mainly driven by reduction of pocket infections.	PM, CRT-D, ICD
Frausing et al. (46)	2021	Retrospective	736/1,207	Absorbable	12 months	Reoperations due to replacement, upgrade or revision	CIED infection incidence 2.3% in AEE recipients vs. 4.1% in controls. (adjusted HR 0.52, 95% CI 0.30-0.90, *P =* 0.021).	CRT-P/D

*AEE, antibiotic-eluting envelope; CIED, cardiac implantable electronic device; CKD, chronic kidney disease; CRT-D, cardiac resynchronization therapy defibrillator; CRT-P, cardiac resynchronization therapy pacemaker; DM, diabetes mellitus; HR, hazards ratio; ICD, implantable cardioverter defibrillator; OAC, oral anticoagulation therapy; PM, pacemaker; RCT, Randomized controlled trial; RRR, relative risk reduction; trsv, transvenous*.

## Cost-Effectiveness

Despite the proven clinical benefit of the AEE, its utilization is associated with an extra cost which might lead to an additional financial burden on the healthcare systems. Economic perspectives of any medical procedure should be subject to a thorough cost-effectiveness analysis (CEA) that serves to assist in decision-making. A widely accepted measure of cost-effectiveness is the incremental cost-effectiveness ratio (ICER) that is most commonly expressed as the cost invested for quality adjusted life years (QALY) gained by implementing the new intervention compared to standard care (49). The decision to reimburse any form of treatment is usually multifactorial and considers numerous factors specific for each country or healthcare system (49). However, decision-making bodies do impose a threshold value for cost effectiveness—the so-called willingness to pay threshold. The World Health Organization has proposed benchmarks based on the gross domestic product per capita in each country (50). According to a joint statement published in 2014 by the American College of Cardiology and the American Heart Association, ICER per QALY gained of < $50 000 was determined to be highly cost-effective, between $50 000 and $150 000 was considered of intermediate cost-effectiveness, and ICER > $150 000 was not considered cost-effective (51). The willingness-to-pay threshold accepted by the UK National Institute of Clinical Excellence is £20 000–30 000, the official threshold accepted in Italy is €25 000–40 000 and ICER < €41 500 per QALY in Germany is considered cost-effective (52).

Cost effectiveness of the AEE has been studied in an early observational study encompassing all ICD and CRT procedures at a single center and calculating additional hospital costs associated with CIED infections (31). At 6 months, the costs associated with CIED infections management exceeded the costs of using AEE as a standard of care by $ 23 863. Another CEA performed in the setting of the UK public healthcare system was based on data from six observational studies of AEE (53). The analysis with a 12-month horizon including the calculated relative risk of 0.163 associated with AEE implantation (84% relative risk reduction) suggested that TYRX™ use was dominant compared to standard of care in ICD and CRT-D and cost effective for CRT-P (ICER £21 768). The AEE was not cost-effective in the patients receiving anti-bradycardia pacemakers (ICER £46 548) suggesting that the economic benefits of AEE are only valid for specific types of devices. Further analysis of this data showed that there is an infection rate threshold for each specific type of devices only above which TYRX™ remains cost-effective. Overall, this study reported that the number needed to treat (NNT) to prevent one device extraction due to CIED infection was 37 while 22 patients needed to be treated for the prevention of one infection-related hospitalization.

**Figure 1:**
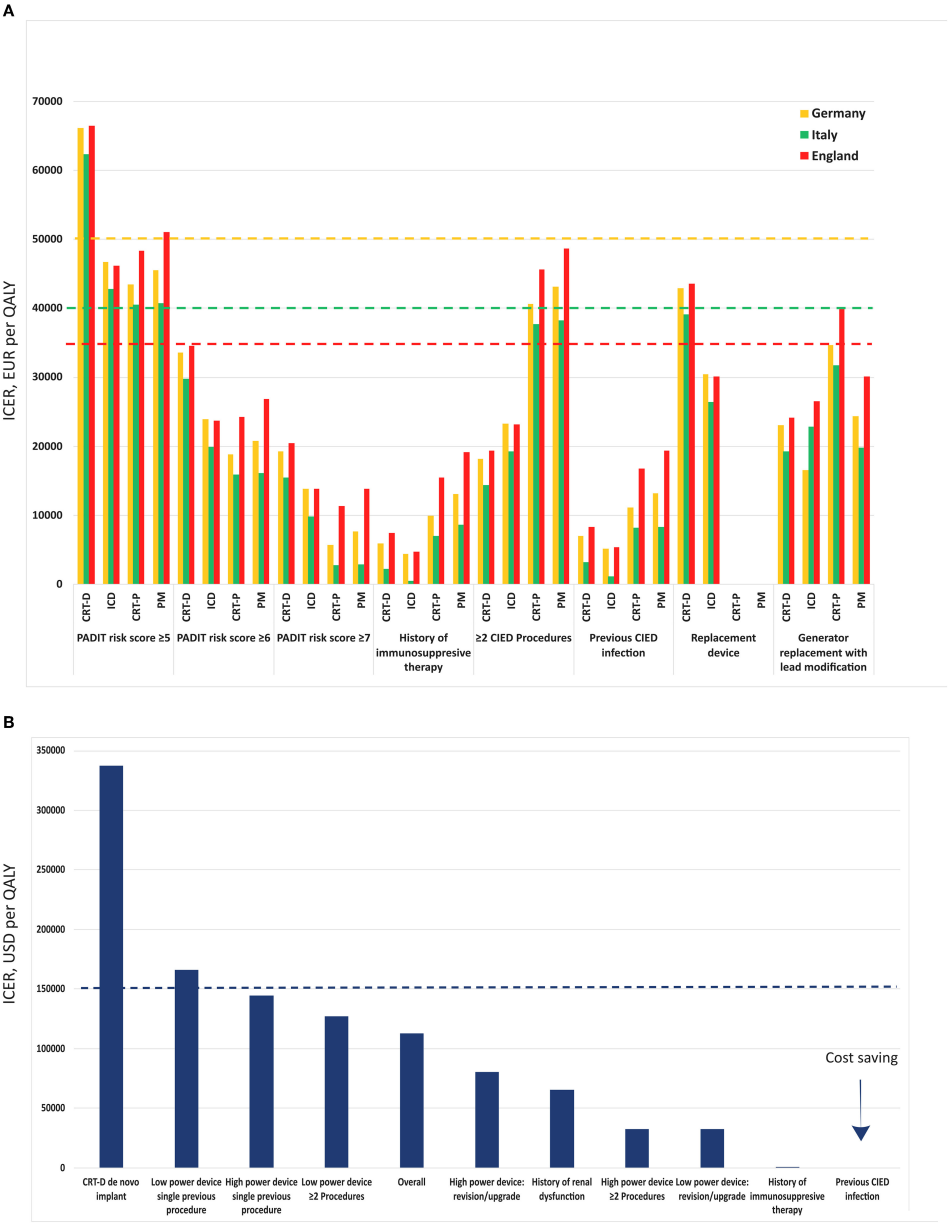
Incremental Cost Effectiveness Ratio (ICER) per Quality Adjusted Life Year (QALY) among different subgroups and based on the population from WRAP-IT trial. Results are shown for Europe **(A)** as reported by Boriani et al. (54) and in US **(B)** as reported by Wilkoff et al. (55). The dashed lines represent the willingness to pay threshold for each country. The values for UK have been recalculated in Euro to facilitate comparability. CIED–cardiac implantable electronic device, CRT-D–cardiac resynchronization defibrillator, CRT-P–cardiac resynchronization pacemaker, ICD–implantable cardioverter defibrillator, PM–pacemaker.

Cost-effective analyses have been performed on the WRAP-IT population as well (Figure 1). A recent CEA in the US healthcare system over a lifetime horizon demonstrated that TYRX™ had an incremental cost effectiveness (55). The use of AEE resulted in 6.925 QALYs at a cost of $37 598 while the standard of care was associated with 6.919 QALYs costing $ 36 929. ICER of TYRX™ was calculated at $112 603 per QALY compared to standard of care. The willingness-to-pay threshold used in the analysis was $150 000 demonstrating overall cost-effectiveness of the AEE. Model iterations with varying infection rates in the standard of care arm demonstrated that TYRX™ is cost saving when the infection rate was ≥4.0% and highly cost-effective with an ICER below $50 000 with infection rates ≥2.0%. The AEE remained cost-effective (ICER < $ 150 000) with an infection rate of ≥1.0% while economic benefits were lost with infection rates <1.0%. Subgroup analysis showed that TYRX™ use in patients with prior CIED infections are cost-saving, while high cost-effectiveness was demonstrated in immunocompromised patients, those with high-power devices, two or more previous procedures, as well as those in revision or upgrade of low-power devices. The use of TYRX™ demonstrated intermediate cost-effectiveness in revision/upgrade or single previous procedure in high-power devices or multiple procedures in low-power devices as well as in patients with a history of renal failure. AEE was not cost-effective in cases of CRT-D *de novo* implants and in cases of single previous procedures in low-power devices. In this study, the NNT to prevent one CIED infection was calculated at 200, probably due to the low infection rates in the studied population.

Another very recent CEA was performed on the WRAP-IT population in the setting of the healthcare systems of several European countries—Italy, Germany and England (54). Based on a decision tree with a lifetime horizon, this analysis uses model inputs from the WRAP-IT (e.g., mortality data, health-related quality of life, probability of CIED infection, etc.) and PADIT trials (probability of CIED infection). In this study, ICER was calculated for each type of CIED in each of the studied countries. Additional analysis of cost-effectiveness based on the PADIT risk score was also included. The willingness-to-pay thresholds considered were €40 000 per QALY in Italy, €50 000 per QALY in Germany, and £30 000 (€35 564) in England. Base-case scenario analysis demonstrated that TYRX™ was cost-effective in each of the three countries in patients with immunosuppressive therapy, those with a previous CIED infection, the ones undergoing generator replacement with lead modification (apart from CRT-P in England), and those having had two or more previous CIEDs and who received a high-power device. The AEE was found to be more cost-effective in patients with higher PADIT risk scores. TYRX™ was shown to be economically efficient in patients with PADIT risk scores ≥ 6 for all device types in all countries. The AEE was not cost-effective for any device type in Italy and England as well as for CRT-D in Germany when the PADIT risk score was estimated at ≥5. Further analyses including risk sharing with the manufacturer demonstrated low direct costs for the healthcare system and thus improving cost-effectiveness.

Contrary to these findings, in the Canadian healthcare system, TYRX™ was recently shown to not be cost-effective for any type of devices in the base-case scenario (56). The calculated ICER per infection prevented was $274 416, which exceeds the willingness-to-pay threshold. When modeling the infection rate in the sensitivity analysis (standard value of 1.2%), the authors found the AEE to be cost-effective at much higher infection rates (>6%). The observed discrepancies with previous publications are likely multifactorial with methodology of the study likely playing a role.

## Conclusion

Cardiac implantable electronic device infections are a major concern in terms of morbidity, mortality, and healthcare costs. Despite the presence of well-defined preventive strategies including antimicrobial agents, the rate of CIED infections continue to rise. Following firm evidence from a large randomized study, supported by confirmative registry data, the AEE has proven to be a major step toward adequate and cost-effective prevention of CIED infections in patients at highest risk of CIED infection.

## Author Contributions

VT drafted the manuscript. CB-L provided critical revision of the manuscript. Both authors contributed to the article and approved the submitted version.

## Conflict of Interest

VT declares receiving direct personal payment from Abbott, Boehringer Ingelheim, Medtronic, and Berlin Chemie Menarini. CB-L has received personal fees from Medtronic, Boston Sci, Bayer, MSD, BMS, Cathprint, Boehringer Ingelheim, Sanofi Aventis.

## Publisher's Note

All claims expressed in this article are solely those of the authors and do not necessarily represent those of their affiliated organizations, or those of the publisher, the editors and the reviewers. Any product that may be evaluated in this article, or claim that may be made by its manufacturer, is not guaranteed or endorsed by the publisher.
